# The double-sided of human leukocyte antigen-G molecules in type 1 autoimmune hepatitis

**DOI:** 10.3389/fimmu.2022.1007647

**Published:** 2022-10-12

**Authors:** Roberto Littera, Andrea Perra, Michela Miglianti, Ignazio S. Piras, Stefano Mocci, Sara Lai, Maurizio Melis, Teresa Zolfino, Cinzia Balestrieri, Maria Conti, Giancarlo Serra, Francesco Figorilli, Davide Firinu, Simona Onali, Laura Matta, Carmen Porcu, Francesco Pes, Daniela Fanni, Cristina Manieli, Monica Vacca, Roberto Cusano, Marcello Trucas, Selene Cipri, Stefania Tranquilli, Stefania Rassu, Federica Cannas, Mauro Giovanni Carta, Marta Anna Kowalik, Erika Giuressi, Gavino Faa, Luchino Chessa, Sabrina Giglio

**Affiliations:** ^1^ Medical Genetics, R. Binaghi Hospital, Sardegna, Italy; ^2^ AART-ODV (Association for the Advancement of Research on Transplantation), Cagliari, Italy; ^3^ Section of Pathology, Oncology and Molecular Pathology Unit, Department of Biomedical Sciences, University of Cagliari, Cagliari, Italy; ^4^ Department of Medical Sciences and Public Health, University of Cagliari, Cagliari, Italy; ^5^ Neurogenomics Division, Translational Genomics Research Institute (TGen), Phoenix, AZ, United States; ^6^ Medical Genetics, Department of Medical Sciences and Public Health, University of Cagliari, Cagliari, Italy; ^7^ Division of Gastroenterology, Azienda di Rilievo Nazionale ed Alta Specializzazione (ARNAS), S. Michele Hospital, Cagliari, Italy; ^8^ Liver Unit, University Hospital, Cagliari, Italy; ^9^ Division of Pathology, Department of Medical Sciences and Public Health, University Hospital San Giovanni di Dio, Cagliari, Italy; ^10^ Department of Pathological Anatomy, Azienda di Rilievo Nazionale ed Alta Specializzazione (ARNAS), S. Michele Hospital, Cagliari, Italy; ^11^ Biomedical Sector, Center for Advanced Studies, Research and Development (CRS4), Cagliari, Italy; ^12^ Centre for Research University Services (CeSAR, Centro Servizi di Ateneo per la Ricerca), University of Cagliari, Monserrato, Italy

**Keywords:** type 1 autoimmune hepatitis, Sardinian population, human leukocyte antigen, HLA-G alleles, soluble HLA-G, plasma cell, anti-HLA-G monoclonal antibodies, HLA-G 3’UTR haplotypes

## Abstract

The immunomodulatory effects of HLA-G expression and its role in cancers, human liver infections and liver transplantation are well documented, but so far, there are only a few reports addressing autoimmune liver diseases, particularly autoimmune hepatitis (AIH).

**Method and materials:**

We analyzed the genetic and phenotypic characteristics of HLA-G in 205 type 1 AIH patients (AIH-1) and a population of 210 healthy controls from Sardinia (Italy).

**Results:**

Analysis of the HLA-G locus showed no substantial differences in allele frequencies between patients and the healthy control population. The HLA-G UTR-1 haplotype was the most prevalent in both AIH-1 patients and controls (40.24% and 34.29%). Strong linkage was found between the HLA-G UTR-1 haplotype and HLA-DRB1*03:01 in AIH-1 patients but not controls (*D’* = 0.92 *vs D’* = 0.50 respectively; P = 1.3x10^-8^). Soluble HLA-G (sHLA-G) levels were significantly lower in AIH-1 patients compared to controls [13.9 (11.6 – 17.4) U/mL *vs* 21.3 (16.5 – 27.8) U/mL; P = 0.011]. Twenty-four patients with mild or moderate inflammatory involvement, as assessed from liver biopsy, showed much higher sHLA-G levels compared to the 28 patients with severe liver inflammation [33.5 (23.6 – 44.8) U/mL *vs* 8.8 (6.1 – 14.5) U/mL; P = 0.003]. Finally, immunohistochemistry analysis of 52 liver biopsies from AIH-1 patients did not show expression of HLA-G molecules in the liver parenchyma. However, a percentage of 69.2% (36/52) revealed widespread expression of HLA-G both in the cytoplasm and the membrane of plasma cells labeled with anti-HLA-G monoclonal antibodies.

**Conclusion:**

This study highlights the positive immunomodulatory effect of HLA-G molecules on the clinical course of AIH-1 and how this improvement closely correlates with plasma levels of sHLA-G. However, our results open the debate on the ambiguous role of HLA-G molecules expressed by plasma cells, which are pathognomonic features of AIH-1.

## Introduction

Autoimmune hepatitis (AIH) is a rare autoimmune disease in which the immune system attacks autologous hepatocytes, causing a chronic/relapsing inflammation of the liver that can lead to cirrhosis and liver failure. In the United States of America, the 5-year prevalence rate of AIH is estimated at 31.2 per 100,000 persons with a higher prevalence among females, the elderly (above 65 years), and individuals of Caucasian ethnicity (1). Studies from Europe indicate an incidence ranging from 0.9 to 2 per 100,000 population per year. A similar incidence has recently been reported in South Korea whereas New Zealand would seem to have an incidence of AIH among the highest reported worldwide (2). The highest point prevalence of the disease has been reported in Alaska (42.9/100,000) (3).

Although it is considered a rare disease, the incidence and prevalence of AIH are growing worldwide, and its complications are the principal indication for liver transplantation for up to 3% and 6% of the pediatric and adult population, respectively. Variants of AIH may occur, with features overlapping with primary biliary sclerosis (PBC) and/or primary sclerosing cholangitis (PSC) occurring in up to 10% of cases (4, 5). While more than 40% of patients develop acute hepatitis, sometimes in the form of fulminant hepatitis, some patients remain asymptomatic (6–8).

To date, the precise pathogenesis of AIH has not been clarified yet. There is strong evidence that environmental factors contribute to the disease, interacting with a predisposing genetic background (2, 9). Since AIH is a polygenic disease, the identification of triggers and genetic predisposing factors has proved challenging, although several studies have pointed to HLAs and TCR genes as potentially playing a role (10, 11). Even though the genetic background of AIH remains elusive, similar to other autoimmune diseases, it is characterized by a loss of immune tolerance that leads to chronic and progressive liver damage and inflammation (12). Among the most important tolerogenic systems, the human leukocyte antigen-G (HLA-G) is the best characterized non-classical major histocompatibility complex (MHC) Class Ib molecule (13, 14). The immunosuppressive effects exerted by this molecule on all types of immune cells are well documented and account for its pivotal role in the induction and maintenance of immune tolerance (15). The *HLA-G* gene maps to the short arm of chromosome 6 in the HLA region (6p21.2-21.3), between the *HLA-A* and *HLA-F* genes. Overall, its gene structure is similar to the classical HLA class I genes, bearing seven introns and eight exons encoding the molecule heavy chain (16, 17). The *HLA-G* gene promoter region comprises a modified enhancer A (enh A), S, and X1 sequence and a few alternative HLA-G transcriptional regulatory elements (18).

Alternative splicing of HLA-G primary transcript generates seven alternative mRNAs encoding membrane-bound (HLA-G1, -G2, -G3, -G4) and soluble (HLA- G5, -G6, -G7) protein isoforms. Soluble HLA-G (sHLA-G) is detectable in both serum and plasma and is mainly produced by monocytes (19, 20). HLA-G expression is regulated by a variety of factors, including genetic variability, post-transcriptional regulation, intracellular and extracellular microenvironmental factors (21). Depending on the cell type and the specific stimulation, some isoforms may be expressed, whereas others may not (22). Compared to the other HLA Class I genes, *HLA-G* displays a relatively low degree of variability with only 102 alleles (https://www.ebi.ac.uk/ipd/imgt/hla/about/statistics/, April 2022) and 35 proteins. Two non-coding regions have been identified as the polymorphic sites with the strongest influence on HLA-G expression: the 5’ upstream regulatory region (5’URR) and the 3’ untranslated region (3’UTR) of the HLA-G gene. These regions also contain a variety of regulatory elements capable of influencing mRNA stability, turnover, splicing and mobility mechanisms (23). According to the results of a worldwide genetic analysis published in 2014, higher or lower secretion of soluble HLA-G (sHLA-G) seems to be associated with haplotypes mapping in the 3’UTR of HLA-G (24, 25). More specifically, certain polymorphic sites located in the HLA-G 3’UTR (14-base pair insertion/deletion, 3003C/T, 3010C/G, 3027A/C, 3035C/T, 3142C/G, 3187A/G, 3196C/G) generate UTR haplotypes (26) which determine different plasma levels of sHLA-G. The UTR-1 haplotype is classified as the highest producer of sHLA-G, whereas UTR-2, UTR-3, UTR-4, and UTR-6 are classified as medium producers and UTR-5 and UTR-7 as low producers (27).

Alongside its limited degree of polymorphism, HLA-G differs from the classical HLA Class I molecules for its restricted pattern of protein expression. Initially, HLA-G expression was considered to be restricted to extravillous cytotrophoblasts (EVT) at the maternal-fetal interface, possibly suppressing immune response in the placenta to protect the fetus from lysis mediated by natural killer (NK) cells (28). Later, it was established that HLA-G protein in healthy non-fetal subjects is found in the thymic medulla, the cornea, proximal nail matrix, beta cells of the islets of Langerhans, mesenchymal stem cells, and erythroid and endothelial precursors (15, 29–32). It is well known that EVT expressing HLA-G has a tolerogenic function at the maternal-fetal interface, where they protect the fetus from destruction by the maternal immune system (33). As reported in **Table 1**, the immunosuppressive role of HLA-G molecules has also been widely explored in numerous inflammatory, immune-mediated and infective conditions as well as in the transplantation setting (25, 39–43, 45, 54). In malignancies, HLA-G expression can directly help tumor cells escape immune surveillance through receptor binding, impairment of chemotaxis, and a mechanism known as trogocytosis (34–37, 44). Recent data in the literature indicate that HLA-G also plays an important role in liver homeostasis and immune response to liver injury and/or cancer (46). These studies demonstrate detectable levels of HLA-G expression in the hepatocytes and biliary epithelial cells of patients with chronic hepatitis B. These patients had higher levels of plasma sHLA-G than healthy controls, albeit different levels of sHLA-G were found according to disease stage (48, 49). Similar results were reported for patients with chronic hepatitis C. In these patients, HLA-G was expressed by mast cells that promote the formation of fibrous septa. Additionally, HLA-G was shown to worsen liver fibrosis by creating a shift in host immune response mechanisms in favor of a TH2 cytokine profile (47, 50).

**Table 1:** Function of human leukocyte antigen-G in physiological and pathological conditions.

Key points	HLA-G functions	Ref.
**Physiological expression in healthy tissues**	HLA-G is known to have tolerogenic properties and a restricted pattern of expression in healthy tissues.	(15, 28–33)
**Autoimmune or inflammatory diseases** **(RA, SLE, MS, IBD, AS)**	Plasma level of sHLA-G correlates with disease activity parameters in RA, SLE, MS, IBD, AS.	(15, 23, 25)
**Cancer** **(LH, MM, GC, CC)**	HLA-G expression can directly help tumor cells escape immune surveillance through receptor binding, impairment of chemotaxis, and a mechanism known as trogocytosis.	(34–38)
**Transplantation** **(HT, HSCT, KT)**	Higher pre-transplant sHLA-G levels were a risk factor for serious infection in HT. sHLA-G high levels are associated with less severe acute and chronic GvHD in allo HSCT. HLA-G 14-bp insertion/deletion polymorphism is associated with susceptibility to rejection in KT.	(39–44)
**Viral infection** **(HCMV, HIV-1)**	HLA-G expression is evidenced during CMV reactivation. HIV-1 uses HLA-G molecule up-regulation to evade immunosurveillance.	(25, 45)
**Physiological expression in liver cells**	HLA-G is not yet fully investigated in liver cells. HLA-G receptors (ILT2, ILT4, KIR2DL4) are present on all immune cells. Microenvironmental factors may influence the production of HLA-G by various cells, including tumor cells, cells of the monocyte lineage, mast cells and biliary epithelial cells.	(46, 47)
**Autoimmune hepatic diseases** **(AIH-1, PBC)**	The beneficial effect needs to be confirmed. HLA-G may induce local temporary immune suppression, counteracting autoimmune inflammatory responses	(46)
**Viral hepatitis infection** **(HBV, HCV)**	Beneficial or detrimental effects according to different situation.	(46–50)
**Tumors** **(HCC, BC)**	HLA-G expression was found in 50.2% (110/219) of the primary HCCs assessed in one study; staining was heterogeneous in these HCCs, but undetectable in the adjacent normal liver tissues.	(46–53)
**Liver transplantation**	HLA-G expression in the liver allograft is associated with a lower frequency of hepatic. An inverse correlation was also found between serum HLA-G concentration and liver function in liver transplant patients.	(46)

sHLA-G, Soluble human leukocyte antigen-G; RA, Rheumatoid arthritis; SLE, Systemic lupus erythematosus; MS, Multiple sclerosis; IBD, Inflammatory bowel disease; AS, Asthma; LH, Hodgkin lymphoma; MM, Multiple Myeloma; GC, Gastric Cancer; CC, Colorectal cancer; HT, HSCT, Hematopoietic stem cell transplantation; KT, Kidney transplantation; HCMV, Human cytomegalovirus; HIV-1, Human immunodeficiency virus-1; AIH-1, Type 1 autoimmune hepatitis; PBC, Primary biliary sclerosis; HBV, Hepatitis B virus infection; HCV, Hepatitis C virus infection; HCC, Hepatocellular carcinoma; BC, Biliary cancer.

Moreover, high levels of sHLA-G were detected in patients diagnosed with hepatocellular carcinoma (HCC), possibly protecting cancer cells from immune attack by inhibiting the activation of immune cells such as CD8-positive T cells, NK cells, B cells, and dendritic cells (DCs) (38). Hence, in HCC, like in other cancers, expression of the tolerogenic HLA-G molecule correlates with a poor prognosis (51–53).

However, despite the recent significant advances in the understanding of HLA-G biology, there are, to the best of our knowledge, no reports investigating the immunomodulatory effects of HLA-G expression and its role in AIH-1. Starting from the evidence that HLA-G molecules have anti-inflammatory effects both in physiological and pathological conditions, this study aimed at investigating the role of HLA-G molecules in type-1 AIH. In particular, we analyzed the genetic and phenotypic characteristics of HLA-G by comparing them with a control population originating from the same geographical area (Sardinia, Italy). Furthermore, we investigated the distribution of HLA-G molecules in liver tissue.

In this study, we show that the role of HLA-G molecules in type-1 AIH is not univocal: on the one side, the immunomodulatory effect of HLA-G molecules improves the clinical course of AIH-1; on the other side, HLA-G expressed in plasma cells may be correlated with more aggressive AIH-1 clinical forms.

## Materials and methods

**Figure 1:**
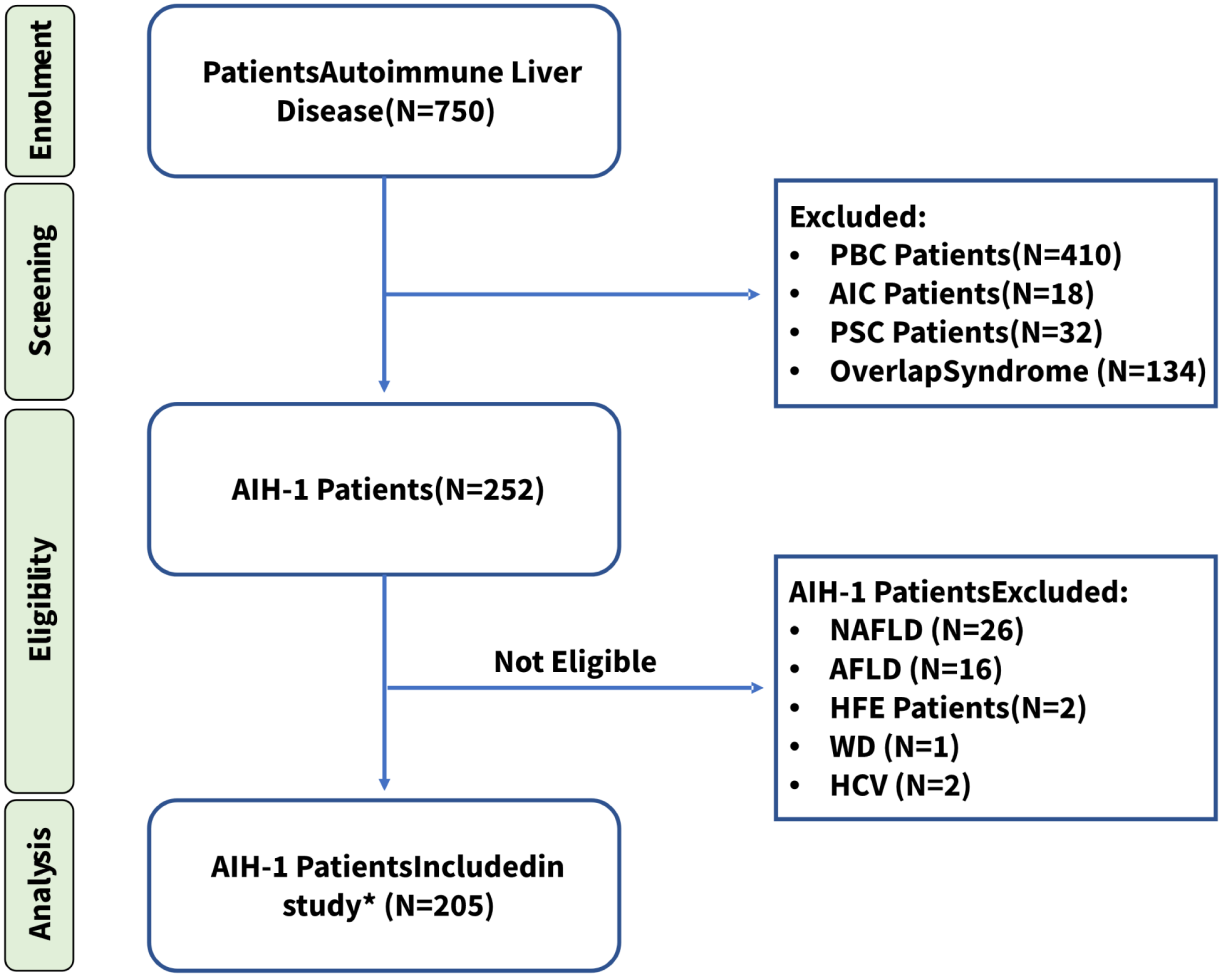
Patient enrollment workflow diagram. * All patients included in the study, were diagnosed according to the International Autoimmune Hepatitis Group (IAIHG) revised scoring system and the American Association for the Study of Liver Diseases (AASLD) Guidelines (55). PBC, Primary biliary cholangitis; AIC, Autoimmune cholangitis; PSC, Primary sclerosing cholangitis; NAFLD, Non-alcoholic fatty liver disease; AFLD, Alcoholic Fatty Liver Disease; HFE, Hereditary hemochromatosis; WD, Wilson Disease; HCV, Hepatitis C. *Fifteen patients (7.3%) tested negative for all AIH-related antibodies but were included in this study because of their high score (≥ 15) according to the IAIHG scoring system at the time of diagnosis.

We recruited a cohort of 205 Sardinian outpatients with AIH type I who were referred to the Center for the Study of Liver Diseases, Department of Medical Sciences and Public Health of the University of Cagliari, and from the Division of Gastroenterology of the ARNAS, S. Michele Hospital (Cagliari, Italy). All patients originated from central-southern Sardinia, were recruited between 02/2014 and 12/2020, and were diagnosed according to the International Autoimmune Hepatitis Group (IAIHG) revised scoring system and the American Association for the Study of Liver Diseases (AASLD) Guidelines (55). To avoid bias, patients with type 2 AIH or overlap syndrome were excluded from the study, as well as patients affected by chronic liver disorders induced by drug or alcohol abuse, fatty liver disease, metabolic disorders, genetic disorders, autoimmune cholangitis, primary biliary cirrhosis, and primary sclerosing cholangitis. All enrolled patients were negative for hepatitis B surface antigen (HbsAg), anti-hepatitis A virus IgM antibody, anti-hepatitis C virus IgG antibody, and anti-hepatitis D virus IgG antibody (**Figure 1**).

The healthy control population corresponded to a panel of 210 individuals of Sardinian origin, going back at least two generations, selected from the regional bone marrow donor registry to represent the genetic background of the Sardinian population (56). This population group appropriately represented the male-to-female ratio and genetic profiles of the population in the central-south geographical areas from where the AIH-1 patients were recruited.

HLA-G alleles and 3’UTR haplotypes were compared between patients and healthy controls. Plasma sHLA-G levels were determined in patients and controls and stratified according to the different HLA-G 3’UTR haplotypes that have been described as influencing the expression of HLA-G (27).

Moreover, a subset of 52 AIH-1 patients whose liver biopsy was adequate for additional immunohistochemical staining, were analyzed to detect the presence of HLA-G in hepatic tissue and to evaluate its possible correlation with the level of hepatic inflammation (mild/moderate or severe).

### Ethics statement

Written informed consent was obtained from each patient or healthy subject included in the study, following the institutional and national ethical standards of the local human research committee. The study protocol, including informed consent procedures, conforms to the ethical guidelines of the Declaration of Helsinki and was approved by the responsible ethics committee (Ethics Committee of the Cagliari University Hospital; date of approval: January 23, 214; protocol number NP/2014/456). Records of written informed consent are kept on file and are included in the clinical record of each patient.

### DNA extraction and HLA typing

Genomic DNA was extracted from peripheral blood mononuclear cells according to standard methods (57). Patients and controls were typed at high-resolution for the alleles at the HLA-A, -B, -C -DR loci and other 13 HLA loci (including HLA-G) using a Next-Generation Sequencing (NGS) method. AlloSeq Tx17 (CareDx), an innovative NGS high-resolution HLA typing solution based on Hybrid Capture Technology (https://caredx.com/products-and-services/transplant-lab-products/hla-typing-solutions/alloseq-tx17/) was used to type all 415 samples (patients and controls). Data analysis was performed with AlloSeq Assign^®^ software (v.1.0.2). Rare or ambiguous HLA alleles were methodically re-typed according to the Sanger sequencing-based typing (SBT) method using the following SBT kits: AlleleSEQR^®^ HLA for the HLA-A, -B, -C and -DRB1 loci (GenDx&GenDx Products, Utrecht, The Netherlands).

### Next-generation sequencing of the HLA-G gene and 3’UTR region

Full-length NGS of the HLA-G gene including the 5’URR, introns, and 3’UTR, was performed using the MiSeq platform (Illumina). The HLA-G gene region extending from positions −1550 to 3404, relative to the start codon, was analyzed using long-range PCR. Specific primers were designed in relation to HLA-G RefSeqGene version NG_029039.1 (NCBI database), as previously described (58). The libraries were prepared starting from 1 ng of PCR product using the Nextera XT DNA Library Preparation Kit. The normalized libraries (4 nM) were pooled and loaded onto V3 flow cells for 600 cycles of bidirectional sequencing in a MiSeq Illumina Sequencer. The automatically generated FASTQ files were then processed with the MiSeq Reporter v2.6 for alignment and variant calling, and VariantStudio Software v3.0 for variant classification (Illumina, Netherlands). The variants were verified individually and then inserted into appropriate electronic spreadsheets for statistical analysis (see below). HLA-G 3’UTR haplotypes were determined by nucleotide sequence variations from +2945 to +3259 nucleotides, using a methodology and a nomenclature described elsewhere (14, 59).

### Soluble HLA-G plasma quantification

Plasma samples were obtained from 205 patients at the time of diagnosis, before treatment and from 210 controls at the time of enrollment. Levels of sHLA-G were determined by the sHLA-G ELISA assay kit (Exbio, Prague, Czech Republic) according to the manufacturer’s instructions. This kit detects both shedding HLA-G-1 and soluble HLA-G5 molecules. Briefly, plasma samples were immediately frozen after separation and stored at -80°C until use. Fifty µl of each sample were diluted 1:80 in the plasma-specific buffer prior to running the HLA-G assay. A six-point calibration curve was obtained using the human native HLA-G protein supplied with the kit. A microplate reader with a 450 nm filter was used to measure the optical density at the end of the reaction. The limit of sensitivity was 0.6 U/ml. All samples were assayed in duplicate.

### HLA-G staining procedure in tissue samples

A subset of 52 AIH-1 patients whose liver biopsy was adequate for additional immunohistochemical stains, were analyzed to detect the presence of HLA-G in tissue. All biopsy specimens had at least 10 portal spaces and were suitable for immunohistochemical analysis.

Tissue samples from liver biopsies were fixed in 10% buffered formalin, routinely processed, and embedded in paraffin. Serial 3-µm-thick sections were obtained from each paraffin block. The serial sections were dewaxed, rehydrated and pretreated for histochemical and immunohistochemical staining. Standard hematoxylin & eosin staining was performed with Mayer hematoxylin and eosin in an aqueous solution. Serial sections were used for HLA-G immunostaining. Briefly, sections were subjected to a 10-min heat-induced epitope retrieval in buffer pH 9.00 (EnVisionTM FLEX Target Retrieval Solution High pH - Dako Denmark A/S, Glostrup, Denmark, Code K8004). Slides were then incubated for 20 min at room temperature with a specific anti-human MHC Class I HLA-G (LifeSpanBioSciences, Inc., Seattle, WA, USA, Code LS-B3734) mouse monoclonal antibody clone 4H84 at 1:100 dilution. Staining procedures were performed with the EnvisionTM FLEX+ (Dako, Code K8002) Detection System and the Autostainer Link 48 instrument according to the manufacturer’s instructions. The immunohistochemical evaluation of HLA-G protein expression in stained liver biopsy specimens was performed by three independent pathologists according to the following scores: 0 points for negative staining (–), 1 for staining <25% of examined cells (+), 2 between 26 and 50% (+), 3 between 51 and 75% (++), 4 between 76 and 100% (+++). The intensity of the staining was not considered for the evaluation of HLA-G expression. The average agreement for the 3 assessed variables among the three pathologists (DF, CM, GF) was 80%, ranging from 70% for Kupffer cells, histiocytes and macrophages to 90% for hepatocytes and plasma cells.

Hepatic inflammation was histologically graded as mild, moderate and severe according to the international grading system of hepatitis activity based on of the presence of portal inflammation, interface hepatitis, and lobular inflammation (60). A correlation between the degree of hepatic inflammation and the HLA-G tissue presence was also investigated.

### Statistical analysis

Clinical and biochemical parameters of the AIH-1 patients were computed using mean and standard deviations (SD) for continuous variables and percentages for categorical data. Ninety-five percent confidence intervals (95% CI) were calculated for all variables. Results for patients were compared by applying the Student’s test and Fisher’s exact test as appropriate.

Comparisons of HLA-G alleles and HLA-G 3’UTR haplotypes between AIH-1 patients and healthy controls were performed using the exact Fisher’s test. P values were adjusted for multiple testing by Bonferroni method accounting for the number of HLA-G 3’UTR haplotypes analyzed. Only corrected P values (Pc) lower than 0.05 were considered to be statistically significant.

Soluble HLA-G plasma levels of AIH-1 patients and healthy controls were expressed using median values, 95% confidence intervals and boxplots. Subgroups of patients and/or controls stratified according to the presence or absence of the UTR-1 haplotype or mild/moderate and severe inflammatory conditions were also evaluated. P values were computed by using the Wilcoxon rank-sum test (61) for data not following a normal distribution. The 95% confidence intervals of the median values were obtained with Hodges-Lehmann statistics (62).

Kendall’s tau rank method (61) was used to evaluate the correlation between sHLA-G levels and other biochemical parameters (ALT, ALP and γ-globulin) in a subset of 52 AIH-1 patients whose liver biopsy was adequate for analysis.

The linkage disequilibrium (LD) between the HLA-G UTR-1 haplotype and the complete or partial Sardinian HLA extended haplotypes was evaluated in AIH-1 patients and healthy controls. The expected frequencies were obtained by multiplying the frequency of the HLA-G UTR-1 haplotype and that of the considered HLA haplotype. The observed and expected frequencies in each sample were compared using the chi-square test.

Linkage disequilibrium was measured by the parameters D (difference between the observed and expected frequencies) and D′ (i.e. D normalized to one: -1 ≤ D′ ≤ 1). D′ was obtained using the normalization formulas proposed by Lewontin (63) for two-loci haplotypes.

In order to compare the linkage disequilibrium in the control and patient group, we computed the P value associated with the Chi-square variable (with two degrees of freedom) given by the difference between the chi-square variables in the two cohorts (with one degree of freedom).

Statistical analysis was performed using R software version 4.2.1 [R Core Team (2022). R: A language and environment for statistical computing. R Foundation for Statistical Computing, Vienna, Austria. URL https://www.R-project.org/].

## Results

### Clinical

The clinical and immunological characteristics of 205 AIH-1 patients are shown in **Table 2**. The mean age at diagnosis was 51.7 (SD 13.3) years and 177 patients (86.3%) were females. Median disease duration at the time of evaluation for this study was 6 years (IQR 6 years). Most of the patients had high titers (64.9%) of antinuclear antibodies (ANA), detected either alone or in combination with anti-smooth muscle antibodies (SMA) (42.9%). Liver kidney microsomal type-1 (LKM-1) and type-3 (LKM-3) antibodies were absent in all samples. Fifteen patients (7.3%) tested negative for all AIH-related antibodies but were included in this study because of their high score (≥ 15) according to the IAIHG scoring system at the time of diagnosis. More than one-third of the patients (35.6%) presented one or more associated autoimmune diseases, including Hashimoto’s thyroiditis, rheumatoid arthritis and mixed connectivitis. One hundred and thirty-eight patients (67.3%) tested positive for HLA-DRB1*03 antigens, and 90 patients (43.9%) tested positive for HLA-DRB1*04 antigens. Most patients (66.8%) had been treated with steroids, either alone or in combination with azathioprine (59.5%), and only eleven patients (5.4%) were non-responders or partial responders to therapy. These results are in line with those reported in previous studies on European and North American populations (1, 64, 65).

**Table 2:** Clinical and biochemical parameters of AIH patients.

Number of patients	Total patients (205)		Pts with severe inflammation in liver biopsy (28)		Pts with severe inflammation vs remaining pts
Gender: n (%)	F: 177 (86.3) M: 28 (13.7)		F: 23 (82.1); M: 5 (17.9)		
	mean ± SD	95% CI	mean ± SD	95% CI	P value*
**Age (yr)**	51.7 ± 13.3	49.6 – 53.8	49.7 ± 15.6	43.4 – 56.0	0.486
**Age at diagnosis (yr)**	43.4 ± 16.1	41.2 – 45.6	38.1 ± 15.9	31.7 – 44.5	0.077
**AST level (IU/L)**	77.2 ± 182.0	52.1 – 102.3	169.5 ± 209.4	84.9 – 254.1	**0.020**
**ALT level (IU/L)**	87.4 ± 212.4	58.2 – 116.7	203.4 ± 251.5	101.8 – 305.0	**0.015**
**ALP level (IU/L)**	145.6 ± 101.1	131.7 – 159.6	236.0 ± 163.6	169.9 – 302.1	**0.004**
**Bilirubin level (μmol/L)**	19.1 ± 19.1	16.4 – 21.7	25.0 ± 43.2	7.6 – 42.5	0.423
**Albumin level (g/dL)**	3.82 ± 0.30	3.78 – 3.86	3.75 ± 0.54	3.53 – 3.97	0.464
**γ-globulin level (g/dL)**	1.69 ± 0.70	1.59 – 1.79	2.04 ± 0.69	1.76 – 2.32	**0.009**
**PT-INR**	1.03 ± 0.08	1.02 – 1.04	1.05 ± 0.13	1.00 – 1.10	0.449
**sHLA-G**	13.86 ± 31.25	11.57 – 17.39	8.83 ± 4.60	6.12 – 14.52	**0.003**
	**n (%)**	**95% CI**	**n (%)**	**95% CI**	P value*
**ANA positivity**	133 (64.9)	58.3 – 71.5	21 (75)	59.9 – 93.9	0.289
**SMA positivity**	88 (42.9)	36.1 – 49.7	15 (53.6)	33.7 – 74.0	0.287
**LKM1 positivity**	0	–	0	–	–
**AMA positivity**	0	–	0	–	**-**
**Autoimmune diseases**	73 (35.6)	29.0 – 42.2	15 (53.6)	33.7 – 74.0	0.066
**Hepatitis B and C**	0	–	0	–	–
HLA-DRB1*03 present	138 (67.3)	60.9 – 73.8	19 (67.9)	50.6 – 87.9	0.954
HLA-DRB1*04 present	90 (43.9)	37.1 – 50.7	10 (35.7)	15.4 – 53.8	0.412
**Prednisone therapy**	137 (66.8)	60.3 – 73.3	17 (60.7)	41.9 – 81.2	0.521
**Azathioprine therapy**	122 (59.5)	52.8 – 66.3	16 (57.1)	37.7 – 77.6	0.811
**UDCA therapy**	21 (10.2)	6.1 – 14.4	4 (15.4)	0.8 – 30.0	0.517
**No reaction to therapy**	11 (5.4)	2.3 – 8.5	2 (7.7)	0.0 – 18.5	0.701

* P values were calculated by comparing the patients with severe inflammation to the remaining AIH-1 patients; the Student’s t-test was used for continuous variables, the exact Fisher’s test for categorical data. P-values marked with bold indicate statistically significant p-values.

Out of the 52 patients whose liver biopsy was adequate for analysis, 44 were females (84.6%), and 8 were males (15.4%). Twenty-eight out of 52 patients (53.8%) presented severe inflammation, while in the remaining 24 patients (46.2%), parenchymal inflammation was mild or moderate. Patients who showed severe inflammation in the liver tissue had higher transaminase (AST, ALT), alkaline phosphatase (ALP), and γ-globulin levels compared to the other AIH-1 patients. Furthermore, in this group of 28 patients, AIH-1 was associated with an increased frequency of other autoimmune diseases [53.8% *vs* 33.0%, P = 0.048]. No other significant differences were found for the remaining clinical and immuno-genetical characteristics (**Table 2**).

### Genetic analysis

The comparison of HLA-G alleles and 3’UTR haplotype frequencies between 205 AIH-1 patients and 210 healthy controls has been reported in **Table 3**. Similar to Northern Europe and Northern America populations, in our AIH-1 patients, the HLA-DRB1*03:01 allele was observed with the highest frequency at the DRB1 locus (**Supplementary Table S1**), reaching a statistically significant difference compared to the control population [38.8% *vs* 21.7%; OR = 2.29 (95% CI 1.67 – 3.15) P = 1.0·10^-7^].

**Table 3:** Extended haplotypes (HLA-G alleles and 3’UTR haplotypes) frequencies in healthy controls and AIH type 1 patients.

Extended haplotypes		210 Healthy controls		205 AIH patients		Controls vs patients	
Alleles	3’UTR haplotypes	2N = 420	*%*	2N = 410	*%*	*P* value	OR (95% CI)
G*01:01:01:01	UTR-1	116	0.2762	138	0.3366	0.059	1.330 (0.989 – 1.788)
G*01:03:01:02	UTR-5	64	0.1524	35	0.0854	0.000	0.519 (0.335 – 0.804)
G*01:01:02:01	UTR-2	50	0.119	65	0.1585	0.100	1.394 (0.938 – 2.073)
G*01:01:03:03	UTR-7	28	0.0667	25	0.061	0.737	0.909 (0.521 – 1.587)
G*01:01:01:08	UTR-1	27	0.0643	27	0.0659	0.927	1.026 (0.591 – 1.782)
G*01:01:22:01	UTR-2	26	0.0619	5	0.0122	0.000	0.187 (0.071 – 0.492)
G*01:04:01:01	UTR-3	23	0.0548	26	0.0634	0.597	1.169 (0.655 – 2.084)
G*01:01:01:05	UTR-4	21	0.05	35	0.0854	0.042	1.773 (1.014 – 3.102)
G*01:06:01:01	UTR-2	15	0.0357	15	0.0366	0.946	1.025 (0.495 – 2.125)
G*01:05N	UTR-2	8	0.019	10	0.0244	0.597	1.288 (0.503 – 3.295)
G*01:04:04	UTR-3	7	0.0167	4	0.0098	0.384	0.581 (0.169 – 2.001)
G*01:01:01:04	UTR-18	7	0.0167	0	0	0.019	0.072 (0.004 – 1.272)
G*01:04:01:01	UTR-10	5	0.0119	2	0.0049	0.268	0.407 (0.078 – 2.109)
G*01:06:01:02	UTR-2	4	0.0095	3	0.0073	0.728	0.767 (0.171 – 3.447)
G*01:01:01:06	UTR-4	3	0.0071	0	0	0.188	0.170 (0.008 – 3.395)
G*01:04:01:02	UTR-3	3	0.0071	0	0	0.188	0.170 (0.008 – 3.395)
G*01:03:01:01	UTR-5	3	0.0071	0	0	0.188	0.170 (0.008 – 3.395)
G*01:01:01:01	UTR-4	2	0.0048	0	0	0.351	0.255 (0.011 – 5.669)
G*01:01:01:04	UTR-6	2	0.0048	10	0.0244	0.018	5.225 (1.138 – 23.994)
G*01:01:01:06	UTR-2	2	0.0048	1	0.0024	0.577	0.511 (0.046 – 5.657)
G*01:06:02:02	UTR-2	1	0.0024	2	0.0049	0.549	2.054 (0.186 – 22.739)
G*01:01:01:01	UTR-3	1	0.0024	0	0	0.693	0.511 (0.017 – 15.272)
G*01:02:02	UTR-2	1	0.0024	0	0	0.693	0.511 (0.017 – 15.272)
G*01:01:01:09	UTR-1	1	0.0024	0	0	0.693	0.511 (0.017 – 15.272)
G*01:04:01:01	UTR-2	0	0	4	0.0098	0.093	8.276 (0.436 – 157.032)
G*01:01:22:01	UTR-10	0	0	3	0.0073	0.173	6.192 (0.309 – 123.990)

% = allele frequencies expressed as decimals.

Analysis of the extended haplotypes (HLA-G alleles and 3’UTR haplotypes) showed no substantial difference in frequencies between patients and healthy controls (**Table 3**). The most prevalent extended haplotypes in both groups were HLA-G*01:01:01:01 with UTR-1, HLA-G*01:01:02:01 with UTR-2, HLA-G*01:03:01:02 with UTR-5 and G*01:01:01:05 with UTR-4 (33.66%, 15.85%, 8.54% and 8.54% in patients *vs* 27.62%, 11.90%, 15.24% and 5.0% in controls). Only HLA-G*01:03:01:02 with UTR-1 showed a lower frequency in patients [8.54% *vs* 15.24%; OR = 0.52 (95% CI 0.33 – 0.82) P = 0.004, Pc = NS]. However, the differences between the frequencies of the HLA-G 3’UTR extended haplotypes in the two groups (AIH-1 patients and controls) never exceeded 6.7%, and the P value was not statistically significant after multiple testing adjustments (P_c_ = NS for all extended haplotypes, except for HLA-G*01:01:22:01 with UTR-2: 1.22% *vs* 6.19%; P = 1.5·10^-4^ and P_c_ = 0.004). HLA-G 3’UTR haplotype frequencies are described in **Table 4**. The UTR-1 haplotype was the most prevalent in both AIH-1 patients and controls (40.24% *vs* 34.29% respectively; P = 0.076). A significant difference in frequency was only found for the UTR-5 haplotype [8.54% *vs* 15.95%, OR = 0.49 (95% CI 0.32 – 0.76); P = 0.001, P_c_ = 0.008].

**Table 4:** Haplotype frequencies observed at the HLA-G 3’UTR polymorphic sites (14bp Ins/Del, 3003C/T, 3010C/G, 3027A/C, 3035C/T, 3142C/G, 3187A/G, 3196C/G) in healthy controls and AIH type 1 patients.

HLA-G 3’UTR	210 healthy controls		205 AIH patients		Controls vs patients		
Haplotypes	2N = 420	*%*	2N = 410	*%*	*P*	OR (95% CI)	*Pc*
UTR-1 (DelTGCCCGC)	144	0.3429	165	0.4024	0.076	1.291 (0.974 – 1.711)	
UTR-2 (InsTCCCGAG)	107	0.2548	105	0.2561	0.965	1.007 (0.737 – 1.376)	
UTR-5 (InsTCCTGAC)	67	0.1595	35	0.0854	0.001	0.492 (0.319 – 0.759)	0.008
UTR-3 (DelTCCCGAC)	34	0.081	30	0.0732	0.674	0.896 (0.538 – 1.494)	
UTR-7 (InsTCATGAC)	28	0.0667	25	0.061	0.737	0.909 (0.521 – 1.587)	
UTR-4 (DelCGCCCAC)	26	0.0619	35	0.0854	0.195	1.414 (0.835 – 2.395)	
UTR-18 (DelTGCCCAC)	7	0.0167	0	0	0.019	0.072 (0.004 – 1.272)	0.152
UTR-10 (DelTCCCGAG)	5	0.0119	5	0.0122	0.969	1.025 (0.294 – 3.566)	
UTR-6 (DelTGCCCAC)	2	0.0048	10	0.0244	0.018	5.225 (1.138 – 23.994)	0.144
UTR-8 (InsTGCCGAG)	0	0	0	0			
UTR-13 (DelTCCTGAC)	0	0	0	0			

% = allele frequencies expressed as decimals.

Because a previous study of Sardinian patients indicates a strong association of AIH-1 with a particular HLA extended haplotype (HLA-A*30:02, C*05:01, B*18:01, DRB1*03:01) (66), the presence of linkage disequilibrium between this HLA extended haplotype and the most prevalent HLA-G3’UTR haplotype (UTR-1) was investigated (**Table 5**). We observed a strong linkage disequilibrium between the UTR-1 haplotype and the aforementioned HLA extended haplotype in patients compared to controls (*D* = 12.97%, *D^1^* = 1 and *D* = 6.49%, *D^1^* = 0.96 respectively; P = 0.002). The most significant difference between AIH-1 patients and controls was found for the linkage disequilibrium between UTR-1 and HLA-DRB1*03:01 (*D* = 7.09%, *D’* = 0.92 and *D* = 46.62%, *D’*= 0.50 respectively; P = 1.3·10^-8^).

**Table 5:** Linkage Disequilibrium between the HLA-G UTR-1 haplotype and HLA alleles and haplotypes in the population control and AIH-1 patient groups.

	210 Healthy controls, 2N = 420 antigens							205 AIH-1 patients, 2N = 410 antigens							Controls vs Patients	
		Observed		Expected					Observed		Expected					P value	
	n	f (%)	n	f (%)	D (%)	D’	*x^2^*	n	f (%)	n	f (%)	D (%)	D’	*x^2^*	
**HLA-G UTR-1 with complete HLA haplotype**															
HLA-G UTR-1, -A*30:02, -B*18:01, -C*05:01, -DRB1*03:01	42	10.00	15	3.51	6.49	0.96	12.72	89	21.71	36	8.74	12.97	1	25.52	0.002	
**HLA-G UTR-1 with partial HLA haplotypes**																
HLA-G UTR-1, -A*30:02, -B*18:01, -C*05:01	48	11.43	17	4.08	7.35	0.94	15.01	89	21.71	36	8.83	12.88	0.98	25.52	0.005	
HLA-G UTR-1, -A*30:02, -B*18:01, -DRB1*03:01	42	10.00	15	3.51	6.49	0.96	12.72	89	21.71	36	8.74	12.97	1	25.52	0.002	
HLA-G UTR-1, -A*30:02, -C*05:01, -DRB1*03:01	41	9.76	15	3.51	6.25	0.93	11.96	87	21.22	36	8.74	12.48	0.96	23.91	0.003	
HLA-G UTR-1, -B*18:01, -C*05:01, -DRB1*03:01	54	12.86	22	5.14	7.72	0.78	13.9	119	29.02	48	11.78	17.24	0.99	36.85	1.0·10^-5^	
HLA-G UTR-1, -A*30:02, -B*18:01	49	11.67	17	4.16	7.51	0.94	15.8	89	21.71	37	9.03	12.68	0.95	24.39	0.014	
HLA-G UTR-1, -A*30:02, -C*05:01	49	11.67	17	4.16	7.51	0.94	15.8	90	21.95	37	9.03	12.92	0.96	25.19	0.009	
HLA-G UTR-1, -A*30:02, -DRB1*03:01	43	10.24	16	3.84	6.40	0.87	12.32	89	21.71	37	8.93	12.78	0.96	24.39	0.002	
HLA-G UTR-1, -B*18:01, -C*05:01	62	14.76	25	5.88	8.88	0.79	16.62	122	29.76	50	12.27	17.49	0.96	37.09	3.6·10^-5^	
HLA-G UTR-1, -B*18:01, -DRB1*03:01	67	15.95	24	5.71	10.24	0.93	21.74	125	30.49	51	12.37	18.12	0.99	38.55	2.2·10^-4^	
HLA-G UTR-1, -C*05:01, -DRB1*03:01	55	13.10	22	5.31	7.79	0.77	14.64	120	29.27	49	11.88	17.39	0.99	36.52	1.8·10^-5^	
**HLA-G UTR-1 with single HLA alleles**																
HLA-G UTR-1, -A*30:02	56	13.33	20	4.73	8.60	0.95	17.72	94	22.93	41	9.91	13.02	0.88	23.98	0.044	
HLA-G UTR-1, -B*18:01	78	18.57	37	8.82	9.75	0.58	16.12	140	34.15	57	13.94	20.21	0.98	44.92	5.6·10^-7^	
HLA-G UTR-1, -C*05:01	72	17.14	29	7.02	10.12	0.75	19.85	125	30.49	53	12.96	17.53	0.91	36.17	2.9·10^-4^	
HLA-G UTR-1, -DRB1*03:01	61	14.52	31	7.43	7.09	0.50	10.27	151	36.83	64	15.61	21.22	0.92	46.62	1.3·10^-8^	

The number of observed and expected haplotypes consisting of the HLA-G UTR-1 haplotype and the complete or partial Sardinian HLA extended haplotypes. The parameter D, the difference between the observed and expected frequencies, is a measure of the linkage disequilibrium. D′ is the parameter D normalized to one. The x^2^ values express the discrepancy between the observed and expected frequencies in each cohort. The P values were obtained from the chi-square variable correspond to the difference between the x^2^ values in the patient and control groups.

n = number of HLA haplotypes or alleles, f = frequencies expressed as percentages.

### Soluble HLA-G dosage

**Figure 2:**
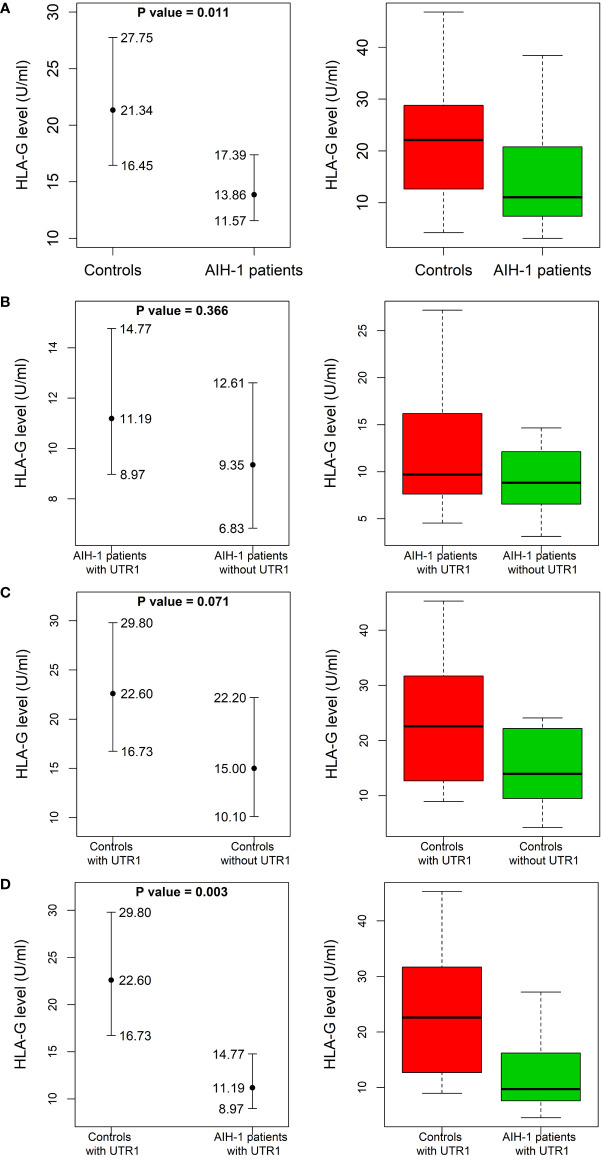
Soluble HLA-G plasma levels in AIH-1 patients and control population. Soluble HLA-G (sHLA-G) plasma levels (U/mL) were compared between AIH-1 patients and the control group **(A)**, two subgroups of the control population stratified according to the presence or absence of the HLA-G UTR-1 haplotype **(B)**, two subgroups of AIH-1 patients stratified according to the presence or absence of the HLA-G UTR-1 haplotype **(C)** and finally between AIH-1 patients and controls who presented the HLA-G UTR 1 haplotype **(D)**. The P values reported in each figure were computed using the Wilcoxon rank sum test. The vertical bars represent the 95% confidence intervals of the median values (full dots). The boxplots show the spread of the soluble HLA-G plasma levels around the median.

Soluble HLA-G (sHLA-G) levels were measured in patients at the time of diagnosis and in healthy controls at the time of enrollment (**Figure 2**).

The levels of sHLA-G were significantly lower in AIH-1 patients than controls [13.9 (11.6 – 17.4) U/mL *vs* 21.3 (16.5 – 27.8) U/mL respectively; P = 0.011] (**Figure 2A**). According to the literature, the HLA-G UTR-1 haplotype is associated with a higher expression of sHLA-G. This prompted us to compare sHLA-G levels between patients and controls stratified according to the presence or absence of the HLA-G UTR-1 haplotype (**Figures 2B–D**). The expression of sHLA-G resulted to be significantly different even when the two subgroups of patients and controls carrying the HLA-G UTR-1 haplotype were compared [11.2 (9.0 – 14.8) U/mL *vs* 22.6 (16.7 – 29.8) U/mL respectively; P = 0.003; **Figure 2D**].

**Figure 3:**
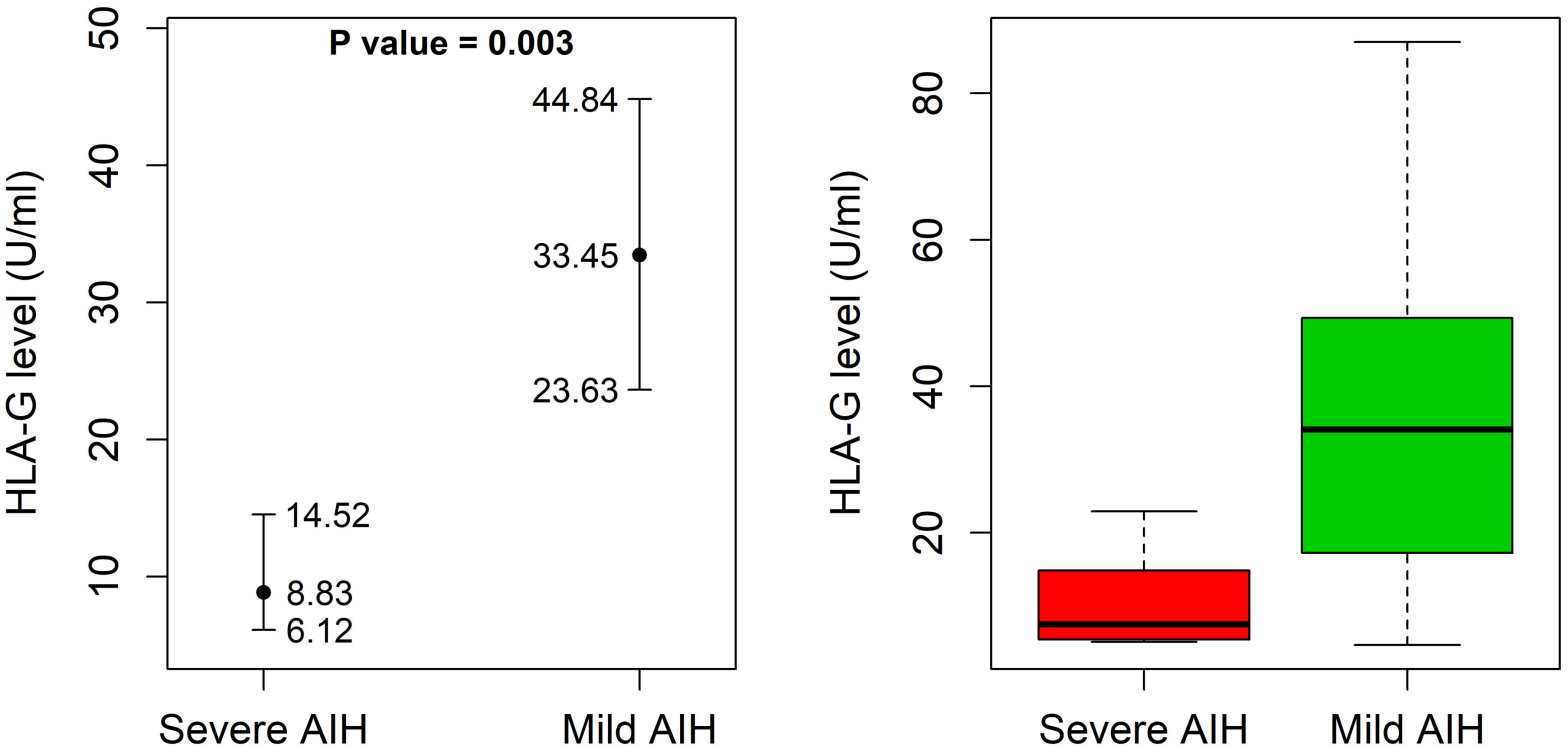
Soluble HLA-G plasma levels in 52 AIH-1 patients for which liver biopsy was available. Soluble HLA-G (sHLA-G) plasma levels (U/mL) were analyzed in 52 AIH-1 patients for which liver biopsy was available, stratified according to the grade of liver inflammation (mild/moderate or severe). The P value reported in the figure was computed using the Wilcoxon rank sum test. The vertical bars represent the 95% confidence intervals of the median values (full dots). The boxplots show the spread of the soluble HLA-G plasma levels around the median.

AIH-1 patients with the UTR-1 haplotype had higher and less variable levels of sHLA-G compared to patients without the UTR-1 haplotype although these differences were not statistically significant [11.2 (9.0 – 14.8) U/mL in UTR-1 positive patients *vs* 9.4 (6.8 – 12.6) U/mL in UTR-1 negative patients; P = 0.37; **Figure 2B**]. Similar results were found in the control group [22.6 (16.7 – 29.8) U/mL in UTR-1 positive controls *vs* 15.0 (10.1 – 22.2) U/mL in negative controls; P = 0.07; **Figure 2C**]. Basal sHLA-G levels measured at the time of diagnosis in the 52 AIH-1 patients with liver biopsy were evaluated for a possible correlation with the severity of liver inflammation (**Figure 3**). It is relevant to note that the 24 patients with mild or moderate inflammatory involvement had much higher sHLA-G levels compared to the 28 patients with severe liver inflammation [33.5 (23.6 – 44.8) U/mL *vs* 8.8 (6.1 – 14.5) U/mL respectively; P = 0.003; **Figure 3**].

Interestingly, as opposed to the biochemical markers ALT, ALP, and γ -globulin, the plasma levels of sHLA-G tend to decrease in severe cases of AIH-1 (**Table 2**). However, correlation analysis based on Kendall’s tau rank did not indicate any statistically significant correlation between sHLA-G levels and ALT, ALP, or globulin levels (τ = -0.17, -0.03, 0.04 and P = 0.10, 0.79, 0.70 respectively).

### Immunohistochemistry

**Figure 4:**
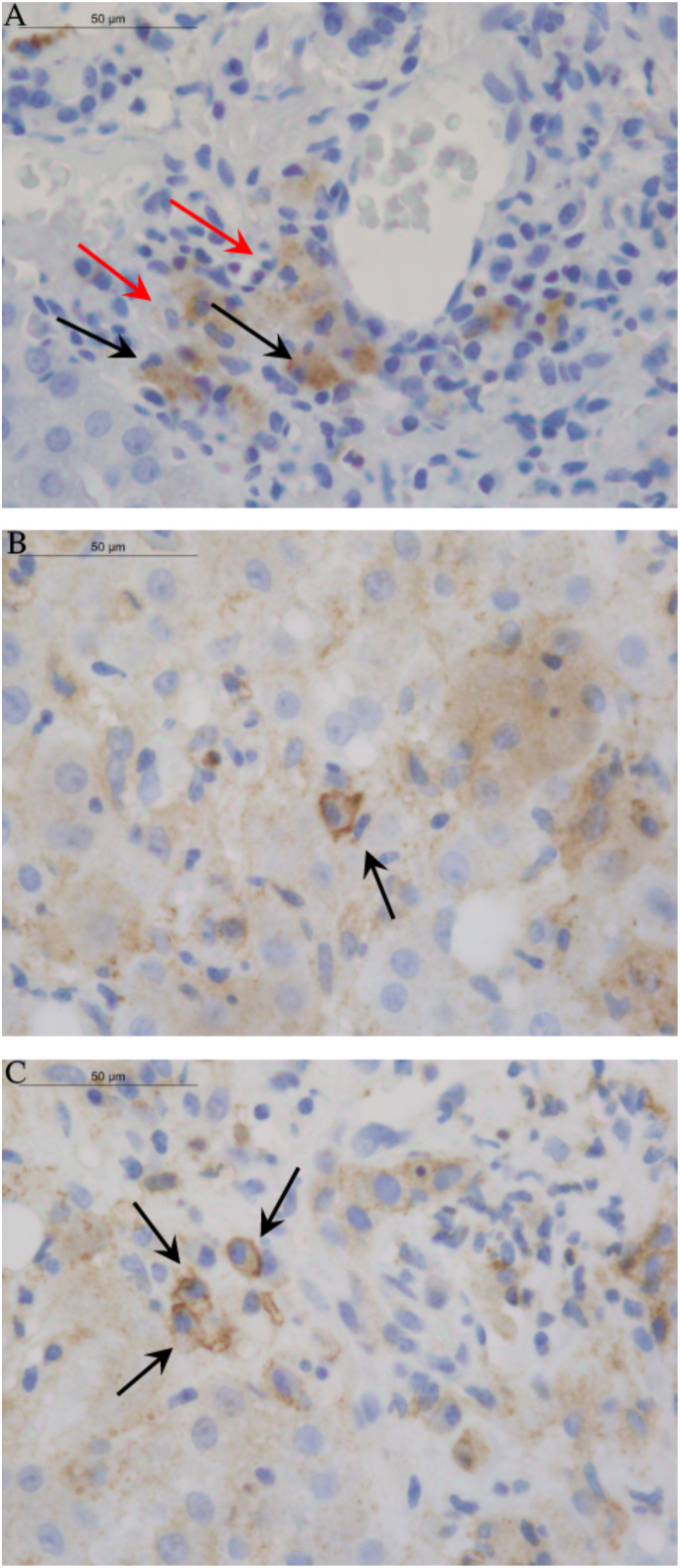
Images of HLA-G distribution in liver tissue. The 4H84 mAb were used to detect HLA-G molecules in different cell types of liver biopsy from AIH-1 patients. **(A)** The picture displayed the absence of HLA-G expression in hepatocytes, cholangiocytes, Kupffer cells and Ito stellate cells. Only Histiocytes/Macrophages (marked by red arrow) and Plasma cells (marked by black arrow) showed widespread HLA-G expression in the cytoplasm. **(B, C)** Single and clustered Plasma cells of AIH-1 patients with severe clinical manifestations showed expression of HLA-G molecules both in the cytoplasm and in the membrane (sHLA-G ≥ 50% and score ≥ ++; see ).

Analysis of liver biopsies from AIH-1 patients did not reveal the expression of HLA-G molecules in the liver parenchyma: hepatocytes, cholangiocytes, Kupffer cells, and Ito stellate cells (**Table 6**). One of the most interesting findings stemming from our study was the widespread expression of HLA-G molecules both in the cytoplasm and membrane of plasma cells labeled with anti-HLA-G monoclonal antibodies (**Figure 4**). This finding was confirmed in the large majority of the examined biopsy specimens [Thirty-six out of 52 samples (69.2%) presented positive plasma cells].

**Table 6:** Analysis of liver biopsies from AIH-1 patients.

Patients ID	AIH Type	Inflammation^&^	Plasma cells			Hepatocytes and Endothelial cells		Kupffer cells			Histiocytes/		
											Macrophages		
			HLA-G (%)	HLA-G (Score)	M and/or C*	HLA-G (%)	HLA-G (Score)	HLA-G (%)	HLA-G (Score)	M and/or C#	HLA-G (%)	HLA-G (Score)	M and/or C#
B. 5390/2014	I	severe	75	+++	M - C	0	–	0	–	–	25	+	C
B. 4307/2015	I	mild	0	–	–	0	–	0	–	–	0	–	–
R. 113/2016	I	severe	100	++++	M - C	0	–	0	–	–	75	+++	C
B. 9458/2015	I	severe	50	++	M	0	–	0	–	–	0	–	–
B. 9171/2014	I	severe	50	++	M - C	0	–	0	–	–	50	++	C
R. 112/2016	I	moderate	25	+	M - C	0	–	0	–	–	50	++	C
B. 6663/2018	I/O^	mild	0	–	–	0	–	0	–	–	0	–	–
R. 96/2017	I/O^	moderate	0	–	–	0	–	0	–	–	0	–	–
B. 7432/2015	I	severe	100	++++	M - C	0	–	25	+	C	25	+	C
B. 3387/2014	I	severe	25	+	M - C	0	–	0	–	–	75	+++	C
B. 7665/2019	I	moderate	<25	+/-	M - C	0	–	0	–	–	<25	+/-	C
B. 7324/2016	I	severe	100	++++	M - C	0	–	0	–	–	75	+++	C
B. 1002/2014	I	moderate	0	–	–	0	–	75	+++	C	75	+++	C
Z. 048/2017	I	severe	75	+++	M - C	0	–	0	–	–	25	+	C
Z. 054/2017	I	mild	0	–	–	0	–	0	–	–	0	–	–
Z. 060/2018	I	severe	100	++++	M - C	0	–	0	–	–	75	+++	C
Z. 062/2016	I	moderate	<25	+/-	M - C	0	–	0	–	–	<25	+/-	C
Z. 063/2014	I	severe	100	++++	M - C	0	–	0	–	–	75	+++	C
Z. 064/2015	I/O	mild	0	–	–	0	–	0	–	–	0	–	–
Z. 065/2017	I	moderate	0	–	–	0	–	75	+++	C	75	+++	C
Z. 067/2016	I	severe	50	++	M	0	–	0	–	–	0	–	–
Z. 071/2015	I	severe	50	++	M - C	0	–	0	–	–	50	++	C
Z. 074/2015	I	moderate	25	+	M - C	0	–	0	–	–	50	++	C
Z. 077/2016	I/O	moderate	0	–	–	0	–	0	–	–	0	–	–
Z. 081/2016	I	severe	100	++++	M - C	0	–	25	+	C	25	+	C
B. 4588/2014	I	severe	75	+++	M - C	0	–	0	–	–	25	+	C
B. 4457/2015	I	mild	0	–	–	0	–	0	–	–	0	–	–
R. 122/2017	I	severe	100	++++	M - C	0	–	0	–	–	75	+++	C
B. 9774/2015	I	severe	50	++	M	0	–	0	–	–	0	–	–
B. 9231/2014	I	severe	50	++	M - C	0	–	0	–	–	50	++	C
R. 142/2016	I	moderate	25	+	M - C	0	–	0	–	–	50	++	C
B. 6466/2018	I/O^	mild	0	–	–	0	–	0	–	–	0	–	–
R. 106/2018	I/O^	mild	0	–	–	0	–	0	–	–	0	–	–
B. 4552/2014	I	severe	100	++++	M - C	0	–	25	+	C	25	+	C
B. 6587/2015	I	severe	25	+	M - C	0	–	0	–	–	75	+++	C
B. 6595/2020	I	mild	<25	+/-	M - C	0	–	0	–	–	<25	+/-	C
B. 5624/2016	I	severe	100	++++	M - C	0	–	0	–	–	75	+++	C
B. 1202/2017	I	moderate	0	–	–	0	–	75	+++	C	75	+++	C
Z. 032/2016	I	severe	75	+++	M - C	0	–	0	–	–	25	+	C
Z. 065/2018	I	mild	0	–	–	0	–	0	–	–	0	–	–
Z. 034/2020	I	severe	100	++++	M - C	0	–	0	–	–	75	+++	C
Z. 078/2014	I	moderate	<25	+/-	M - C	0	–	0	–	–	<25	+/-	C
Z. 073/2015	I	severe	100	++++	M - C	0	–	0	–	–	75	+++	C
Z. 032/2014	I/O	mild	0	–	–	0	–	0	–	–	0	–	–
Z. 090/2018	I	mild	0	–	–	0	–	75	+++	C	75	+++	C
Z. 027/2016	I	severe	50	++	M	0	–	0	–	–	0	–	–
Z. 087/2016	I	severe	50	++	M - C	0	–	0	–	–	50	++	C
Z. 064/2016	I	mild	25	+	M - C	0	–	0	–	–	50	++	C
Z. 098/2018	I/O	moderate	0	–	–	0	–	0	–	–	0	–	–
Z. 099/2018	I	severe	100	++++	M - C	0	–	25	+	C	25	+	C
Z. 023/2020	I	severe	25	+	M - C	0	–	0	–	–	75	+++	C
Z. 082/2017	I	severe	25	+	M - C	0	–	0	–	–	75	+++	C

^&^ = Hepatic inflammation was histologically graded as mild, moderate and severe according to the international grading system of hepatitis activity based on the presence of portal inflammation, interface hepatitis, and lobular inflammation (60).

M = cell membrane localization of HLA-G molecule; C = cytoplasmic localization of HLA-G molecule;

* = diffuse cytoplasmic distribution of HLA-G molecule; # = granular cytoplasmic distribution of HLA-G molecule.

^ = I/O: Type I AIH patients with minimal inflammatory involvement of the intrahepatic biliary tract.

The biopsies of the examined cases were taken at the time of diagnosis. Immunohistochemical evaluation of HLA-G protein expression according to the scores: for negative staining (–), for staining <25% of examined cells (+), between 26 and 50% (+), between 51 and 75% (++), between 76 and 100% (+++).

Another important finding was the greater expression of HLA-G molecules in the plasma cells of patients with severe liver inflammation and serious clinical manifestations of AIH-1 (sHLA-G ≥ 50% and score ≥ ++, except for two patients). On the contrary, no AIH-1 patients with mild hepatic inflammation had plasma cells characterized by the presence of cytoplasmic and/or membrane HLA-G molecules. No other element of the typical inflammatory infiltrate was positive for anti-HLA-G antibodies.

## Discussion

In this study, we investigated the role of HLA-G molecules in type-1 AIH. Specifically, we analyzed the genetic and phenotypic characteristics of HLA-G comparing a group of AIH-1 patients with a control population originating from the same geographical area (Sardinia, Italy). Additionally, we investigated the distribution of HLA-G molecules in liver tissue in a subset of patients who underwent liver biopsy. Previous studies have described the pathways of HLA-G-induced tolerance and the role that these molecules play in autoimmune diseases (67, 68), such as rheumatoid arthritis (69, 70) and systemic lupus erythematosus (71, 72), but so far, no studies have investigated the impact of HLA-G expression on autoimmune hepatitis. This may partly be due to the difficulty of recruiting a large enough cohort of AIH-1 patients, as well as a genetically homogeneous control population. Even today, the homogeneity of the Sardinian Island population continues to offer an ideal study ground for the analysis of both complex genetic traits and diseases, providing information that is difficult to obtain in other populations.

First of all, this study demonstrates a considerable overlap between the clinical and immuno-genetical parameters observed in Sardinian AIH-1 patients and others from mainland Italy. Moreover, similar results have been reported for patients from North America (1, 64).

In particular, as described in our AIH-1 patients, the HLA-DRB1*03 (*03:01) allele is the main susceptibility factor for AIH-1 in white northern Europeans and North Americans, although a high frequency has also been reported for the HLA-DRB1*04 allele (65).

It is important to note that in our group of AIH-1 patients, the HLA-DRB1*04 susceptibility antigen was represented by HLA-DRB1*04:05 instead of HLA-DRB1*04:01 observed in white North American and Northern European AIH-1 patients (65).

Another interesting observation is that the Sardinian population showed similar frequencies of HLA-G alleles and 3’UTR haplotypes between AIH-1 patients and controls.

Although the HLA-G UTR-1 haplotype was more frequent in AIH-1 patients, it did not reach statistical significance in comparison with controls. Interestingly, this haplotype was in strong linkage disequilibrium with the extended haplotype HLA-A*30:02, -C*05:01, -B*18:01, -DRB1*03:01 (**Table 4**), and even more so with the three-loci haplotype HLA-C*05:01, -B*18:01, -DRB1*03:01 (P = 1.0 x 10^-5^) which we found strongly associated with AIH-1 patients in a previous study (66). HLA-C*05:01, -B*18:01, -DRB1*03:01 is an ancestral extended haplotype probably deriving from the paleo-Mediterranean population that lived in the western regions of the Mediterranean up until the Mesolithic period (56). Infectious diseases such as brucellosis (73, 74), genetic drift, besides the peculiar structure of this haplotype (75), are likely to account for the high frequency of this haplotype in the Sardinian population. However, the high ability of this extended haplotype to activate the immune system has the disadvantage of increasing the risk of serious autoimmune disease in carriers of this haplotype (66, 75). Most probably, the strong LD between the HLA-G UTR-1 polymorphism and the HLA-C*05:01, -B*18:01, -DRB1*03:01 extended haplotype has the function of counterbalancing the reactivity of the immune system triggered by this HLA extended haplotype, by promoting expression of HLA-G molecules with a tolerogenic function.

Similar to healthy controls, we found that sHLA-G levels were higher in AIH-1 patients who express UTR-1 (**Figures 2B, C**).

Overall, AIH-1 patients exhibited sHLA-G levels significantly lower than those observed in the control population (P = 0.011), which is similar to what has been reported for other autoimmune diseases, such as Systemic Lupus Erythematosus (76), Multiple Sclerosis (77), and rheumatoid arthritis (69). Expression of these tolerogenic HLA-G molecules seems to exert a beneficial tolerogenic effect on AIH-1, as evidenced by higher levels of sHLA-G (**Figure 3**) found in the plasma of patients with mild/moderate hepatic inflammation compared to severe forms. Also, the histological picture of patients with high sHLA-G levels was much less severe than that of patients with low plasma levels of sHLA-G, whose biopsies revealed strong inflammatory activity with marked lymphocytic infiltrate.

It is interesting to note that sHLA-G levels show an opposite trend to the biochemical markers ALT, ALP and γ-globulin which tend to increase in severe inflammatory forms of AIH-1.

However, the correlation analysis did not show any statistically significant correlation between sHLA-G levels and ALT, ALP and γ-globulin levels (τ = -0.17, -0.03, 0.04). Therefore, this seems to indicate that sHLA-G levels are independent of these biochemical markers, thus making it useful for discriminating between mild and severe forms of AIH-1. Future studies involving large case series are needed to confirm this result, which was obtained by analyzing a limited number of biopsy samples.

Analysis of liver biopsies from AIH-1 patients did not reveal expression of HLA-G molecules in the liver parenchyma (hepatocytes, cholangiocytes, Kupffer cells, Ito stellate cells). This finding is similar to what has already been described in the literature for the liver of healthy subjects (www.proteinatlas.org). The presence of plasma cells in liver parenchyma represents the hallmark of diagnosis in AIH-1. One of the most interesting findings emerging from our study was the positivity of plasma cells with anti-HLA-G monoclonal antibodies, observed in the large majority of the examined bioptic specimens. Interestingly, no other element of the typical inflammatory infiltrate was positive for anti-HLA-G antibodies (**Figure 4**). Diffuse expression of sHLA-G in the cytoplasm of the plasma cells suggests that these cells not only produce the autoantibodies leading to the inflammatory reaction in AIH-1, but contemporarily produce sHLA-G, probably to protect themselves against the immune cell attacks for which they are responsible. This hypothesis is supported by the fact that in the examined histological samples, the increased expression of HLA-G molecules by plasma cells is directly associated with more severe clinical manifestations (**Table 6**) and a marked inflammatory infiltrate in the hepatic parenchyma.

In conclusion, this is the first study that thoroughly investigated the role of HLA-G molecules in type-1 autoimmune hepatitis. In particular, our results demonstrate how the immunomodulatory effect of HLA-G molecules has a positive impact on the clinical course of AIH-1 and how this improvement closely correlates with plasma levels of sHLA-G. We highly recommend similar and larger studies on genetically different populations to confirm the positive prognostic value of plasma levels of sHLA-G in AIH-1. Based on our findings, it should be possible to hypothesize the use of these molecules in the near future for therapy in autoimmune disorders, particularly considering that these molecules are already considered promising and relevant targets for cancer immunotherapy (78). Meanwhile, our results open the debate on the role of HLA-G molecules expressed by plasma cells, that are ‘pathognomonic’ of AIH-1. In myeloma patients, HLA-G transfer from tumor plasma cells to T cells *via* trogocytosis was associated with a poor clinical outcome (36). It is possible that also in AIH-1, the plasma cells utilize the transfer of HLA-G molecules through the release of exosomes and/or *via* trogocytosis to counteract the immune cell activity of the surrounding microenvironment. The effect of HLA-G plasma cell expression on AIH-1 clinical outcome might therefore be highly dependent on the expression pattern of HLA-G isoforms. Perhaps HLA-G expressed in plasma cells may reflect a high level of epigenetic deregulation and/or genetic mutation, correlating with the more aggressive clinical forms of AIH-1.

## Data availability statement

The datasets presented in this study can be found in online repositories. The names of the repository/repositories and accession number(s) can be found below: https://www.ncbi.nlm.nih.gov/, PRJNA859661.

## Ethics statement

This study was reviewed and approved by Ethics Committee of the Cagliari University Hospital. The patients/participants provided their written informed consent to participate in this study.

## Author contributions

All authors contributed to the article and approved the submitted version.

## Funding

The research performed in this report falls within the institutional responsibilities of the investigators of the participating centers, all of which pertain to the Italian National Public Health Service. The authors received no specific funding for this work. Grant funding (2021-290) was received from the “Fondazione di Sardegna”. The funders had no role in study design, data collection and analysis, decision to publish, or preparation of the manuscript.

## Acknowledgments

We are grateful to the Fondazione di Sardegna for supplying part of the reagents and services necessary for this study. We wish to thank Anna Maria Koopmans for her help in coordinating research activities, professional writing assistance and preparation of the manuscript.

## Conflict of interest

The authors declare that the research was conducted in the absence of any commercial or financial relationships that could be construed as a potential conflict of interest.

## Publisher’s note

All claims expressed in this article are solely those of the authors and do not necessarily represent those of their affiliated organizations, or those of the publisher, the editors and the reviewers. Any product that may be evaluated in this article, or claim that may be made by its manufacturer, is not guaranteed or endorsed by the publisher.
