# Supplementary material for: The double-sided of human leukocyte antigen-G molecules in type 1 autoimmune hepatitis
Source: Front Immunol. 2022 Oct 12;13:1007647. doi: 10.3389/fimmu.2022.1007647 (PMC9597675; doi:10.3389/fimmu.2022.1007647)
Supplement: Supplementary file 1 [file Table_1.docx]

**Supplementary table S1: Observed HLA alleles and haplotypes in AIH-1 patients and controls**

|  | **210 healthy controls, 2N = 420 antigens** | | **205 AIH-1 patients, 2N = 410 antigens** | | **Controls vs patients** | |
| --- | --- | --- | --- | --- | --- | --- |
| **HLA alleles and haplotypes** | **2N** | **(%)** | **2N** | **(%)** | **OR (95% CI)** | **P value** |
| HLA-A*30:02 | 58 | 13.81 | 101 | 24.63 | 2.040 (1.428-2.915) | 0.000074 |
| HLA- B*18:01 | 108 | 25.71 | 142 | 34.63 | 1.531 (1.135-2.064) | 0.005 |
| HLA- C*05:01 | 86 | 20.48 | 132 | 32.20 | 1.844 (1.346-2.526) | 0.000125 |
| HLA-DRB1*03:01 | 91 | 21.67 | 159 | 38.78 | 2.290 (1.687-3.109) | < 0.00001 |
| HLA-DRB1*04:05 and *04:03 | 52 | 12.38 | 91 | 22.20 | 2.019 (1.391-2.929) | 0.000182 |
| HLA- A*30:02, -B*18:01 | 51 | 12.14 | 92 | 22.44 | 2.093 (1.441-3.041) | 0.000086 |
| HLA- A*30:02, -C*05:01 | 51 | 12.14 | 92 | 22.44 | 2.093 (1.441-3.041) | 0.000086 |
| HLA- A*30:02, -DRB1*03:01 | 47 | 11.19 | 91 | 22.20 | 2.264 (1.544-3.319) | 0.000021 |
| HLA- B*18:01, -C*05:01 | 72 | 17.14 | 125 | 30.49 | 2.120 (1.525-2.947) | < 0.00001 |
| HLA- B*18:01, -DRB1*03:01 | 70 | 16.67 | 126 | 30.73 | 2.218 (1.592-3.090) | < 0.00001 |
| HLA- C*05:01, -DRB1*03:01 | 65 | 15.48 | 121 | 29.51 | 2.287 (1.629-3.210) | < 0.00001 |
| HLA- A*30:02, -B*18:01, -C*05:01, | 50 | 11.90 | 90 | 21.95 | 2.081 (1.428-3.033) | 0.000111 |
| HLA- A*30:02, -B*18:01, -DRB1*03:01 | 43 | 10.24 | 89 | 21.71 | 2.431 (1.640-3.602) | < 0.00001 |
| HLA- A*30:02, -C*05:01, -DRB1*03:01 | 43 | 10.24 | 89 | 21.71 | 2.431 (1.640-3.602) | < 0.00001 |
| HLA- B*18:01, -C*05:01, -DRB1*03:01 | 63 | 15.00 | 120 | 29.27 | 2.345 (1.666-3.301) | < 0.00001 |
| HLA- A*30:02, -B*18:01, -C*05:01, -DRB1*03:01 | 43 | 10.24 | 89 | 21.71 | 2.431 (1.640-3.602) | < 0.00001 |
| HLA-G UTR-1 | 144 | 34.29 | 165 | 40.24 | 1.291 (0-974-1.711) | 0.076 |
